# Influence of cyber-victimization and other factors on depression and anxiety among university students in Bangladesh

**DOI:** 10.1186/s41043-023-00469-0

**Published:** 2023-11-06

**Authors:** Tareq Rahman, Md. Mahin Hossain, Nurun Nahar Bristy, Md. Zahidul Hoque, Md. Moyazzem Hossain

**Affiliations:** 1 BRAC James P Grant School of Public Health, BRAC University, Dhaka, 1213 Bangladesh; 2 Department of Statistics, Jahangirnagar University, Savar, Dhaka, 1342 Bangladesh

**Keywords:** Anxiety, Cyberbullying, Cyber-victimization, Depression, University students

## Abstract

**Background and objectives:**

Cyber-victimization is closely linked with mental health problems such as anxiety, depression, etc., and has become a growing concern among university students in Bangladesh. In the era of globalization, smart gadgets, the internet, and other online resources are readily available, and these tools and devices have now become the primary method for cyberbullying. The authors aim to explore the impacts of cyber-victimization and other factors on anxiety and depression among university students in Bangladesh.

**Methods:**

The primary data for this cross-sectional study were collected using a well-structured questionnaire. This study employs three widely used scales such as cyberbullying inventory, general anxiety disorder-7 (GAD-7), and patient health questionnaire-9 (PHQ-9). Descriptive statistics and multivariable logistic regression analyses are carried out to identify the factors associated with depression and anxiety among university students in Bangladesh.

**Results:**

Findings depict that the prevalence of depression and anxiety among university students was 52.5% and 44.0%, respectively. Depressed respondents were considerably more likely to have difficulty sleeping (*p* < 0.001), spend more time on social media (*p* = 0.002), have suicidal thoughts (*p* < 0.001), and have a high cyber-victimization score (*p* < 0.001) compared to non-depressed respondents. In comparison with non-anxious respondents, anxious respondents were significantly more likely to: have sleeping difficulties (*p* < 0.001); spend more time on social media (*p* = 0.031); have suicidal thinking (*p* < 0.001); and have a comparatively high cyber-victimization score (*p* < 0.001). Multivariable logistic regression analysis identified that a one-unit increase in the cyber-victimization score results in a 1.24 times higher chance of experiencing depression (AOR: 1.24, 95% CI 1.17–1.31, *p* < 0.001), and a one-unit increase in the cyber-victimization score results in a 1.23 times higher chance of experiencing anxiety (AOR: 1.23, 95% CI 1.17–1.30, *p* < 0.001).

**Conclusion:**

University students are struggling with cyberbullying, which can lead to depression and anxiety levels. Promoting more cyberbullying awareness is necessary since failing to do so could result in a sustained or increased prevalence of anxiety and depression levels among students, which could have disastrous repercussions.

## Introduction

The utilization of the internet, electronic gadgets, and social media can offer numerous advantages to individuals; however, it can create chances for new forms of harmful interactions such as cyberbullying [1–3]. A complicated and multifaceted phenomenon, cyberbullying is described as "willful and repeated harm inflicted through the use of computers, cell phones, or other electronic devices" and can take several forms including flaming (online altercations using harsh and sarcastic messaging); harassment (delivering a constant stream of nasty and disrespectful messages); cyberstalking (the use of threats or other forms of intimidation that are repeated, severe, and demeaning); denigration (distributing false information, paying for online rumors, or creating online rumors about someone in an effort to harm their reputation or relationships); impersonation (pretending to be someone else and then sending and uploading anything to put the victim in danger, cause difficulties for them, or harm their reputation or relationships with others); outing (disclosing a person’s private information, contents, or embarrassing details); trickery (posting online someone else's unpleasant or private information); and exclusion (deliberately and cruelly excluding someone from a group on the internet) [4, 5]. Due to its unique characteristics, cyberbullying is more harmful than traditional bullying, leading to more social and emotional problems, such as isolation and emotion regulation problems, than victims of traditional bullying [6–10].

In the era of globalization, smart gadgets are readily available, and by using the internet and other online resources, students are able to maintain social connections with one another. Due to the ease of access to social media and technology, these same tools and devices have now become the primary method for cyberbullying [11, 12]. Cyberbullying is said to have a significant negative effect on a student's life. A study pointed out that there is a connection between cyberbullying and adverse psychological and physical difficulties [11]. Additionally, studies have revealed that victims of cyberbullying experience detrimental effects on their physical and psychological well-being [13]. For example, depression, loneliness, anxiety, low self-esteem, self-harm, and even suicidal behavior have been connected with cyberbullying victimization [14].

The previous studies pointed out that the prevalence of depression was relatively high in different countries, including India (males: 27.8% and females: 49.1%) [15], Pakistan (40.9%) [16], Hong Kong (68.5%) [17], Sri Lanka (36%) [18], Saudi Arabia (43%) [19], and Egypt (63.3%) [20]. Meanwhile, rates are lower in Australia (21.8%) [21] and the USA (23%) [22]. Moreover, a higher level of anxiety is present among students in Pakistan (74.2%) [16] and Hong Kong (54.4%) [17] compared to 28.5% in Australia [21] and 25% in the USA [16, 22]. A previous study highlighted that anxiety (64.8%) and depression (54.3%) were both prevalent among Bangladeshi medical students, respectively [23]. A more recent survey of university students in Bangladesh found that the prevalence of depression was 52.2%, and the prevalence of anxiety was 58.1% (across all years as opposed to just 1st year students) [24]. A study also reported that more than 40% of university students suffer from severe anxiety [25].

As it is relatively simple to access social media and technology, cyberbullying is a way for students to violently show their rage [11]. Researchers pointed out that 49% of school pupils and 26% of university students had experienced cyberbullying in some capacity, putting students or young people at risk of getting familiar with this type of harassment [26]. Despite the fact that it is a relatively new problem, experts from all around the world have documented the prevalence and adverse effects of cyberbullying [27–33]. It was frequently observed that victims of cyberbullying had extreme discomfort, emotional instability, social anxiety, and even attempted suicide or other significant bodily injuries [33]. The existing literature depicts that there is a linkage between anxiety and depression with cyberbullying. However, in the context of Bangladesh, in recent years, the country has witnessed a significant surge in internet usage, paralleled by an increasing inclination toward using digital platforms, including social media, for educational purposes. Consequently, the prevalence of cyberbullying victims has also seen a concurrent rise, leading to psychological challenges among students [34]. According to a report named "Bangladesh Cybercrime Trend 2023" published by the Cybercrime Awareness Foundation (CAF) in 2022, abusive posts on social media and cyberbullying accounted for 52.21% of all online offenses that were reported, with university students making up the majority of those victims [35]. Cyberbullying has an adverse impact on the mental health of its victims, leading to increased levels of depression and anxiety [36]. There is a scarcity of research exploring the association between anxiety and depression with cyberbullying among university students in Bangladesh. In order to fill up this research gap, the authors aimed to ascertain the connection between cyberbullying and the level of anxiety and depression among university students in Bangladesh who have experienced cyberbullying. Moreover, this study may offer some advice for victims of cyberbullying and the cyberbullying protection system that will aid in lessening cyberbullying in Bangladesh, particularly at the university level. The authors believed that the findings of this study can persuade everyone that cyberbullying poses a threat to university students' mental health and daily lives.

## Methods and materials

The primary data for this cross-sectional study were collected using a well-structured Google Forms questionnaire from March 1, 2023, to March 30, 2023. The participants were selected through a convenience sampling technique from social media platforms such as Facebook and WhatsApp, following the eligibility criterion of being a current university student in Bangladesh. Prior to start the survey, consent was taken from the participants. As a web-based survey, the students were advised to complete the questionnaire with honesty and integrity, following eligibility requirements, consenting to voluntary participation, and only submitting it once. In total, 491 students (142 men and 349 women) participated in the study.

### Measures

The widely used cyberbullying inventory (CBI) comprised two highly similar forms: The first form contained 16 items related to cyberbullying, while the second form included 18 items focused on cyber-victimization. In a study involving 183 Turkish public school students, the cyber-victimization form of CBI demonstrated a commendable internal consistency coefficient of 0.88 [37]. The GAD-7 is a broadly recognized screening instrument designed to assess generalized anxiety. The validation of the Bangla version of the GAD-7 was conducted in prior studies assuring the suitability for research in Bangladesh [38–40]. The depression assessment screening tool known as PHQ-9 has also been validated in the previous studies conducted in Bangladesh [39, 41, 42].

### The cyberbullying inventory (CBI)

This study employs a form consisting of 18 items for cyber-victimization, rated on a 4-point Likert scale (1—never; 2—once or twice; 3—three to five times; and 4—more than five times) to assess the current status of victims. The score range for cyber-victimization (CV) is from 18 to 72, with higher scores indicating a greater degree of victimization [43]. Examples of items from the CBI include “Someone spread rumors about me on the internet” and “Someone stole my password to access my inbox” [37]. This study demonstrates an excellent internal consistency of the CBI, with a Cronbach's alpha of 0.967.

### General anxiety disorder-7 (GAD-7)

The GAD-7 is a self-report scale designed to assess the likelihood of anxiety disorder cases, comprising of seven items that ask participants how frequently they were troubled by certain things over a 2-week period (such as “Worrying too much about different things, becoming easily annoyed or irritable, feeling afraid as if something awful might happen”) [44]. Respondents provided their answers using a 4-point scale ranging from 0 "not at all" to 3 "nearly every day," with the results being summed and displayed on a scale of 0–21 [45]. Scores greater than 10 were considered indicative of positive screening for generalized anxiety disorder in the current study [46]. The GAD-7 exhibited an excellent level of internal consistency, with a Cronbach's alpha of 0.937.

### Patient health questionnaire-9 (PHQ-9)

The patient health questionnaire (PHQ) is a self-administered tool designed to diagnose common mental disorders, comprising of nine items that ask participants, how frequently they were bothered by certain things over the past 2 weeks such as “Trouble falling or staying asleep, or sleeping too much, Feeling bad about yourself—or that you are a failure or have let yourself or your family down, thoughts that you would be better off dead or of hurting yourself in some way” [47]. The respondents used a 4-point scale to provide their answers, where 0 indicated "not at all" and 3 represented “nearly every day.” The scores were then added together and displayed on a scale from 0 to 21 individuals who scored 10 or higher on the PHQ-9 which were classified as depressed individuals [46]. In the study, PHQ-9 confirmed excellent reliability with a Cronbach's alpha of 0.932.

### Statistical analysis

First, the data are saved in Microsoft Excel, then it is transferred to IBM SPSS Statistics 23.0 and R 4.2.2 for further investigation. The SPSS software is used to analyze the general characteristics of students by employing descriptive statistics. The reliability and correlation of the factors with depression and anxiety are also examined through SPSS. In this study, multivariable logistic regression analyses are carried out using R 4.2.2.

## Results

In this study, a total of 491 students were included, the majority of whom (71.1%) were female. Additionally, 42.4% of the students fell within the age range of 21–23 years, 62.9% were from urban areas, and 72.9% were undergraduates. About 32.8% of the students stated that they spent 2–4 h on social media, whereas 55.2% of the participants reported that they spent 4-h daily. Sleeping problems were reported by 66.4% of the participants. The proportions of students who were victimized by friends, relatives, academic personnel, and unknown individuals were 18.5%, 6.5%, 6.5%, and 68.4%, respectively. Among the participants, shockingly 17.3% reported that they were thinking about suicide because of excessive victimization. The average cyber-victimization score of the participants was 32.11 with a standard deviation of 13.90 (Table 1).

**Table 1:** Background characteristics of the participants

Variables	Participants *n* (%)	Depression			Anxiety		
		No	Yes	*P* value	No	Yes	*P* value
		*n* (%)	*n* (%)		*n* (%)	*n* (%)	
Gender							
Male	142 (28.9)	89 (38.2)	53 (20.5)	< 0.001	104 (37.8)	38 (17.6)	< 0.001
Female	349 (71.1)	144 (61.8)	205 (79.5)		171 (62.2)	178 (82.6)	
Age group							
< 21	91 (18.5)	32 (13.7)	59 (22.9)	0.001	21 (7.6)	70 (32.4)	< 0.001
21–23	208 (42.4)	92 (39.5)	116 (45.0)		134 (48.7)	74 (34.3)	
24–26	139 (28.3)	86 (36.9)	53 (20.5)		96 (34.9)	43 (19.9)	
> 26	53 (10.8)	23 (9.9)	30 (11.6)		24 (8.7)	29 (13.4)	
Residence							
Rural	182 (37.1)	75 (32.2)	107 (41.5)	0.033	96 (34.9)	86 (39.8)	0.264
Urban	309 (62.9)	158 (67.8)	151 (58.5)		179 (65.1)	130 (60.2)	
Relationship status							
Single	348 (70.9)	155 (66.5)	193 (74.8)	0.001	193 (70.2)	155 (71.8)	0.001
Married	86 (17.5)	57 (24.5)	29 (11.2)		57 (20.7)	29 (13.4)	
In a relationship	57 (11.6)	21 (9.0)	36 (14.0)		25 (9.1)	32 (14.8)	
Education							
Undergraduate	358 (72.9)	165 (70.8)	193 (74.8)	0.320	211 (76.7)	147 (68.1)	0.032
Graduate	133 (27.1)	68 (29.2)	65 (25.2)		64 (23.3)	69 (31.9)	
Skin tone							
Fair	200 (40.7)	104 (44.6)	96 (37.2)	0.010	109 (39.6)	91 (42.1)	0.073
Brown	266 (54.2)	124 (53.2)	142 (55.0)		157 (57.1)	109 (50.5)	
Dark	25 (5.1)	5 (2.1)	20 (7.8)		9 (3.3)	16 (7.4)	
Sleeping problem							
Yes	326 (66.4)	179 (76.8)	147 (57.0)	< -0.001	224 (81.5)	102 (47.2)	< 0.001
No	165 (33.6)	54 (23.2)	111 (43.0)		51 (18.5)	114 (52.8)	
Social media account							
≤ 2	324 (66.0)	163 (70.0)	161 (62.4)	0.078	192 (69.8)	132 (61.1)	0.043
> 2	167 (34.0)	70 (30.0)	97 (37.6)		83 (30.2)	84 (38.9)	
Time spend social media							
< 2 h	59 (12.0)	36 (15.5)	23 (8.9)	0.002	42 (15.3)	17 (7.9)	0.031
2–4 h	161 (32.8)	87 (37.3)	74 (28.7)		91 (33.1)	70 (59.7)	
> 4 h	271 (55.2)	110 (4.2)	161 (62.4)		142 (51.6)	129 (59.7)	
Mostly victimized by							
Friends	91 (18.5)	36 (15.5)	55 (21.3)	< 0.001	46 (16.7)	45 (20.8)	< 0.001
Relatives	32 (6.5)	16 (6.9)	16 (6.2)		6 (2.2)	26 (12.0)	
Academic personnel	32 (6.5)	2 (0.9)	30 (11.6)		2 (0.7)	30 (13.9)	
Unknown individual	336 (68.4)	179 (76.8)	157 (60.9)		221 (80.4)	115 (53.3)	
Suicidal thinking for excessive victimization							
No	406 (82.7)	217 (93.1)	189 (73.3)	< 0.001	260 (94.5)	146 (67.6)	< 0.001
Yes	85 (17.3)	16 (6.9)	69 (26.7)		15 (5.5)	70 (32.4)	
CV score							
(Mean ± SD)	32.11 ± 13.90	24.99 ± 5.35	38.54 ± 15.97	< 0.001	25.98 ± 6.18	39.92 ± 16.80	< 0.001

The prevalence of depression and anxiety was 52.5% and 44.0%, respectively, with significant age and gender differences. Depressed respondents were considerably more likely to have difficulty sleeping (*p* < 0.001), spend more time on social media (*p* = 0.002), have suicidal thoughts (*p* < 0.001), and have a high cyber-victimization score (*p* < 0.001) compared to non-depressed respondents. In comparison with non-anxious respondents, anxious respondents were significantly more likely to: have sleeping difficulties (*p* < 0.001); spend more time on social media (*p* = 0.031); have suicidal thinking (*p* < 0.001); and have a comparatively high cyber-victimization score (*p* < 0.001) (Table 1).

**Fig. 1:**
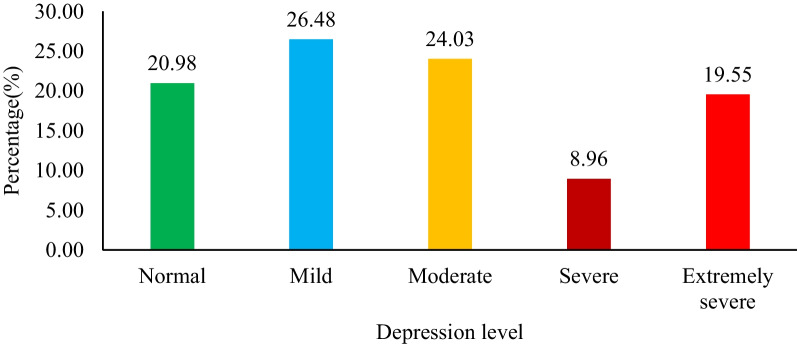
Prevalence of depression levels among university students

In accordance with the PHQ-9 scores, 26.48% of university students had mild depression, 24.03% had moderate depression, 8.96% had severe depression, and 19.55% had extremely severe depression (Fig. 1).

**Fig. 2:**
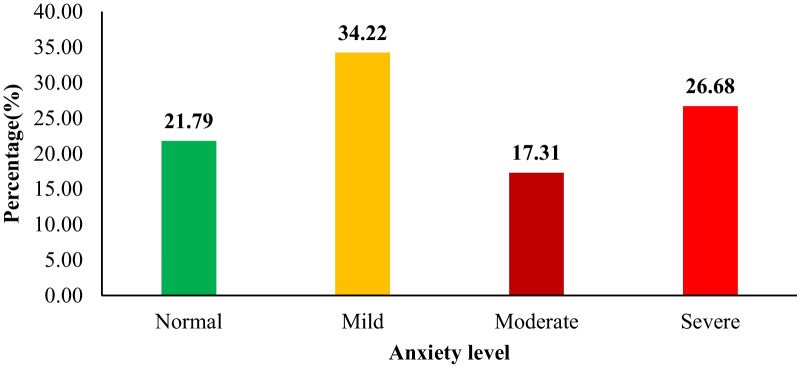
Prevalence of anxiety levels among university students

On the other hand, based on the GAD-7 scale, 21.79% of the students had normal anxiety, 34.22% had mild anxiety, 17.31% had moderate anxiety, and 26.68% had severe anxiety (Fig. 2).

Table 2 depicts that in the unadjusted model, female students had 2.39 times higher odds of experiencing depression (COR: 2.39, 95% CI 1.60–3.57, *p* < 0.001) in comparison with male students. Additionally, the unadjusted model demonstrated that individuals aged between 24 and 26 years had a 47% lower likelihood of being depressed (COR: 0.47, 95% CI 0.25–0.90, *p* < 0.022) than those above 26 years of age, and those residing in rural areas had 1.49 times greater odds of having depression (COR: 1.49, 95% CI 0.03–2.16, *p* = 0.034) than those living in urban areas. Findings revealed that students who experienced sleeping difficulties had a 2.50 times higher chance of developing depression (COR: 2.50, 95% CI 1.69–3.70, *p* < 0.001) than those who did not have such issues. Similarly, individuals who spent more than 4-h daily on social media had a 2.29 times greater likelihood of experiencing depression (COR: 2.29, 95% CI 1.29–4.08, *p* = 0.005) compared to those who spent less than 2 h. Students who were victimized by academic personnel had a 9.82 times higher chance of experiencing depression than those who were victimized by their friends (COR: 9.82, 95% CI 2.21–43.64, *p* = 0.003). There is a statistically significant positive association between cyber-victimization score and the likelihood of depression. For each unit increase in cyber-victimization score, the odds of being depressed increase by a factor of 1.13 (COR: 1.13, 95% CI 1.10–1.16, *p* < 0.001). However, the findings of the adjusted model indicate that female students who experienced cyber-victimization had 18.27 times higher odds of having depression (AOR: 18.27, 95% CI 7.56–44.14, *p* < 0.001) in comparison with male students who were victims of cyber-victimization, while considering the effects of other confounding variables. It also reveals that, when all other factors remain constant, a one-unit increase in the cyber-victimization score results in a 1.24 times higher chance of experiencing depression (AOR: 1.24, 95% CI 1.17–1.31, *p* < 0.001) (Table 2).

**Table 2:** Results of regression analysis of factors associated with depression

Variables	Unadjusted model		Adjusted model^a^	
	COR (95% CI)	*p* value	AOR (95% CI)	*p* value
Gender				
Male	Reference		Reference	
Female	2.39 (1.60–3.57)	< 0.001	18.27 (7.56–44.14)	< 0.001
< 21	1.41 (0.71–2.83)	0.328	0.99 (0.33–2.94)	0.978
21–23	0.97 (0.53–3.13)	0.913	1.05 (0.40–2.80)	0.92
24–26	0.47 (0.25–0.90)	0.022	0.24 (0.08–0.71)	0.01
> 26	Reference		Reference	
Residence				
Rural	1.49 (0.03–2.16)	0.034	1.70 (0.94–3.09)	0.081
Urban	Reference		Reference	
Relationship status				
Single	0.73 (0.41–1.23)	0.278	0.53 (0.22–1.26)	0.152
Married	0.28 (0.15–0.58)	0.001	0.07 (0.03–0.24)	< 0.001
In a relationship	Reference		Reference	
Education level				
Undergraduate	Reference		–	**–**
Graduate	0.82 (0.55–1.22)	0.321		
Skin tone				
Fair	Reference		Reference	
Brown	1.24 (0.90–1.80)	0.25	1.76 (1.00–3.08)	0.047
Dark	4.33 (1.57–12.00)	0.005	4.51 (0.86–23.61)	0.074
Sleeping problem				
Yes	2.50 (1.69–3.70)	< 0.001	1.42 (0.81–2.48)	0.219
No	Reference		Reference	
Social media account				
≤ 2	Reference		Reference	
> 2	1.40 (0.96–2.05)	0.078	1.15 (0.68–1.95)	0.6
Time spend in social media				
< 2 h	Reference		Reference	
2–4 h	1.33 (0.76–2.45)	0.356	1.37 (0.52–3.58)	0.524
> 4 h	2.29 (1.29–4.08)	0.005	1.16 (0.68–1.95)	0.734
Mostly victimized by				
Friends	Reference		Reference	
Relatives	0.66 (0.29–1.47)	0.305	0.13 (0.03–0.67)	0.014
Academic personnel	9.82 (2.21–43.64)	0.003	7.32 (1.24–43.09)	0.028
Unknown individual	0.57 (0.36–0.92)	0.021	0.76 (0.39–1.51)	0.438
CV score	1.13 (1.10–1.16)	< 0.001	1.24 (1.17–1.31)	< 0.001

Table 3 displays that in the unadjusted model, female students had 2.95 times higher odds of experiencing anxiety (COR: 2.95, 95% CI 1.86–4.37, *p* < 0.001) in comparison with male students. Additionally, the unadjusted model demonstrated that individuals age less than 21 years are 2.76 times higher (COR: 2.76, 95% CI 1.33–5.71,* p* = 0.006), those aged between 21 and 23 years have a 46% lower chance of being anxious (COR: 0.46, 95% CI 0.25–0.84, *p* = 0.012), while individuals between 24 and 26 years old have a 37% lower likelihood of being anxious (COR: 0.37, 95% CI 0.19–0.71, *p* = 0.003) compared to those over 26 years old. Students who were married had a 40% lower chance of experiencing anxiety than those who were in a relationship (COR: 0.40, 95% CI 0.20–0.79, *p* = 0.009) and who completed graduation had a 1.55 times greater likelihood of experiencing anxiety than those who were still undergraduates (COR: 1.55, 95% CI 1.04–2.31, *p* = 0.032). Findings also revealed that students who experienced sleeping difficulties had a 4.91 times higher chance of developing anxiety (COR: 4.91, 95% CI 3.28–7.56, *p* < 0.001) than those who did not have such issues. Individuals who had more than two social media accounts had a 1.47 times greater likelihood of experiencing anxiety (COR: 1.47, 95% CI 1.01–2.14, *p* = 0.044) compared to those who had less than two social media accounts. Similarly, individuals who spent more than 4-h daily on social media had a 2.24 times greater likelihood of experiencing anxiety (COR: 2.24, 95% CI 1.23–4.14, *p* = 0.010) compared to those who spent less than 2 h.

**Table 3:** Results of regression analysis of factors associated with anxiety

Variables	Unadjusted model		Adjusted model^a^	
	COR (95% CI)	*p* value	AOR (95% CI)	*p* value
Gender				
Male	Reference		Reference	
Female	2.95 (1.86–4.37)	< 0.001	83.14 (13.26–279.13)	< 0.001
Age group				
< 21	2.76 ( (1.33–5.71)	0.006	15.45 (3.29–77.31)	0.001
21–23	0.46 (0.25–0.84)	0.012	0.49 (0.11–2.10)	0.337
24–26	0.37 (0.19–0.71)	0.003	0.075 (0.02–0.30)	< 0.001
> 26	Reference		Reference	
Residence				
Rural	1.23 (0.85–1.78)	0.246	–	–
Urban	Reference			
Relationship status				
Single	0.62 (0.36–1.10)	0.105	0.99 (0.37–2.68)	0.997
Married	0.40 (0.20–0.79)	0.009	0.13 (0.04–0.491)	0.002
In a relationship	Reference		Reference	
Education level				
Undergraduate	Reference		Reference	
Graduate	1.55 (1.04–2.31)	0.032	5.75 (2.14–15.43)	0.001
Skin tone				
Fair	Reference		Reference	
Brown	0.33 (0.57–1.20)	0.329	1.48 (0.4–2.98)	0.269
Dark	2.13 (0.90–5.05)	0.086	0.18 (0.05–0.69)	0.012
Sleeping problem				
Yes	4.91 (3.28–7.56)	< 0.001	4.40 (2.34–8.29)	< 0.001
No	Reference		Reference	
Social media account				
≤ 2	Reference		Reference	
> 2	1.47 (1.01–2.14)	0.044	1.50 (0.81–2.78)	0.197
Time spend in social media				
< 2 h	Reference		Reference	
2–4 h	1.90 (1.00–3.62)	0.051	2.35 (0.83–6.65)	0.108
> 4 h	2.24 (1.23–4.14)	0.010	1.04 (0.42–2.56)	0.931
Mostly victimized by				
Friends	Reference		Reference	
Relatives	4.43 (1.67–11.78)	0.003	8.24 (1.07–63.64)	0.043
Academic personnel	15.33 (3.46–67.98)	< 0.001	9.26 (1.34–64.16)	0.024
Unknown individual	0.53 (0.33–0.85)	0.008	0.32 (0.14–0.73)	0.007
CV score	1.11 (1.08–1.13)	< 0.001	1.23 (1.17–1.30)	< 0.001

Students who were victimized by relatives had a 4.43 times higher chance of experiencing anxiety than those who were victimized by their friends (COR: 4.43, 95% CI 1.67–11.78, *p* = 0.003) and who were victimized by academic personnel had a 15.33 times higher chance of experiencing anxiety than those who were victimized by their friends. There is a statistically significant positive association between cyber-victimization score and the likelihood of anxiety. For each unit increase in cyber-victimization score, the odds of being anxious increase by a factor of 1.11 (COR: 1.11, 95% CI 1.08–1.13, *p* < 0.001). However, in the adjusted model, sleeping problems, education level, and marital relationship status were found to be significant independent predictors of anxiety (*p* < 0.05). Female students who experienced cyber-victimization had 83.14 times higher odds of having anxiety (AOR: 83.14, 95% CI 13.26–279.13, *p* < 0.001) in comparison with their counterparts who were victims of cyber-victimization while considering the effects of other confounding variables. It also reveals that, when all other factors remain constant, a one-unit increase in the cyber-victimization score results in a 1.23 times higher chance of experiencing anxiety (AOR: 1.23, 95% CI 1.17–1.30, *p* < 0.001) (Table 3).

## Discussion

Mental health problems among university students have grown to be a serious problem for the development of global mental health policies and university campus health services [48, 49]. Being a victim of cyberbullying has significant impacts on developing mental health issues [1, 2]. Although there is limited research concerning mental health problems among university students, there is no prior study that gives a linkage between cyber-victimization and anxiety or depression in university students from Bangladesh. Consequently, the present research aimed to address this knowledge gap by examining the occurrence of anxiety and depression among university students who had experienced cyber-victimization. This study assessed the relationship between bullying victimization and symptoms of depression and anxiety among Bangladeshi university students after controlling for potential confounders and demographic variables. The outcomes of this study showed that bullying victimization was in fact a significant risk factor for disclosing symptoms of depression and anxiety which is consistent with research showing that bullying victimization has consequences for the mental health of those who are bullied [50–52]. This study found that university students who have been the victims of cyberbullying are more likely to have depression and anxiety. This finding is supported by a previous study that has shown that university students frequently experience these conditions [24]. The results showed that girls reported greater levels of cyber-victimization and were likely to be experiencing depression and anxiety more than boys [53]. One possible explanation could be connected to the concept of cyber-victimization, which is frequently a form of indirect bullying that girls are more likely to face [54]. The findings show that those with darker skin are more likely to experience cyberbullying and experience more depression and anxiety which also supports the findings from another study [55]. Female students believed that lighter skin increases the likelihood of self-esteem [56].

The findings of this study revealed that the prevalence of depression (52.5%) was greater, and the prevalence of anxiety was lower (44.0%) compared to a more recent survey of 590 undergraduate university students in Bangladesh, where the prevalence of depression was 52.2%, and the prevalence of anxiety was 58.1% [24]. A higher prevalence of depression is observed among students in Pakistan, Sri Lanka, Saudi Arabia, Australia, and the USA and a lower prevalence of depression in Hong Kong and Egypt. However, a higher prevalence of anxiety is observed among students in Australia and America and a lower prevalence of anxiety among students in Hong Kong and Pakistan [16–22]. The study suggests that students are more often victimized by unknown individuals. If the perpetrator is someone from the academic staff, the likelihood of developing depression and anxiety is higher than if the perpetrator is a friend or relative. The study also showed that married individuals are less likely to be victimized by cyberbullying with respect to individuals who are single or in a relationship, which was also similar to a prior study comprised of 284 college students in the USA [57]. Though it is found that spending more time on social media has an increased chance of developing depression and anxiety, no significant evidence was found to justify that it causes cyber-victimization-related depression and anxiety which differs from the findings from another study [57]. Also, sleeping problem due to cyberbullying has shown significant results for developing anxiety but not depression according to the results found from the study. This study showed that the depression and anxiety caused by cyber-victimization have a significant impact on developing suicidal thinking in line with prior work [58].

## Strengths and limitations

The main strength of this study is its novelty. This study is based on cross-sectional and self-reported data, which may introduce biases. Causal inference is not possible because of its cross-sectional nature. The findings may change because of sampling variability. The sample size used in this study is relatively small and may not be representative of all university students in the country. In the future, study will use a large, diverse sample that represents all universities in the country, which may produce more insightful findings.

## Conclusion

University students are increasingly struggling with cyberbullying, which can lead to MHPs such as depression and anxiety. Moreover, in extreme cases, it can even lead to suicide. Education on cyberbullying is crucial for detecting and preventing such behavior, promoting internet safety, and reducing cyberbullying on university campuses. The findings revealed a high prevalence of depression and anxiety, and it also identified certain risk variables that are strongly linked to the level of depression and anxiety among university students in Bangladesh. Promoting more cyberbullying awareness is necessary since failing to do so could result in a sustained or increased occurrence of this problem among students, which could have disastrous consequences.

## Data Availability

The primary data used to support the findings of this study are available from the corresponding author upon request.
